# Sustainability Assessment of Water Resources Use in 31 Provinces in China: A Combination Method of Entropy Weight and Cloud Model

**DOI:** 10.3390/ijerph191912870

**Published:** 2022-10-08

**Authors:** Yi Zhang, Wenwen Xue, Yingnan Wen, Xianjia Wang

**Affiliations:** 1 School of Economics and Management, Hubei University of Technology, Wuhan 430068, China; 2 School of Economics and Management, Wuhan University, Wuhan 430072, China

**Keywords:** sustainability of water resources use, cloud model, indicator system for assessment, assessment system, China

## Abstract

As a fundamental and strategic resource, water is a crucial controlling element of ecosystem and natural environment and it plays an irreplaceable role in maintaining and promoting the sustainable development of the economy and society. To achieve the sustainable development of society, the economy and ecology, it is necessary to assess and improve the sustainability of water resources use. Based on the Human–Resource–Nature approach, this paper constructed an indicator system for the sustainability assessment of water resources use (ISSAWRU) in China from three perspectives: water resources condition, socio-economy and ecological environment. A five-level hierarchy of assessment indicators was established. Based on the entropy weight method and the cloud model which took both fuzziness and randomness into account, this paper established an entropy-cloud-based assessment model for the sustainability assessment of water resources use in 31 provinces in China in 2019. The assessment results were compared with results obtained by the TOPSIS method to test their reliability. Finally, a comprehensive and in-depth analysis of the sustainability of water resources use in China was conducted. According to the results, water resources per capita had a weighting of 0.306 and the greatest impact on the sustainable use of water resources. In addition, water structure, agricultural water use efficiency, forest coverage, and so on, had a significant impact on the sustainable use of water resources in China. The overall level of sustainability of water resources use in 31 provinces in China was not high—42% of the regions have unsustainable water resources use and there was a clear spatial distribution trend. The sustainability level of water resources use was higher in the southeast and economically developed regions. Therefore, each region should develop measures to guarantee water security based on the local conditions. This research helps policy makers to figure out the contributing factors associated with sustainability of water resources use and to set relevant rules and regulations to promote the use of water resources in a sustainable way.

## 1. Introduction

With changes in climate and in the prosperity of the economy, people have increasing demand for water resources. Therefore, the imbalance between water supply and demand, as well water pollution, have become prominent problems. Water pollution causes damage to the water ecosystem and the healthiness of human beings, plants and animals, exacerbating water scarcity. The issue of water resources has long been a global problem. More and more countries are beginning to develop appropriate strategies for sustainable development of water resources [1]. In 1977, the UN Water Council announced that water could cause a severe social crisis in the near future, in the manner of the oil crisis. In 2019, the World Resources Institute released a report pointing out that 17 countries around the world had been in a state of extreme water scarcity, 12 of which were in the Middle East and North Africa, and nearly 50% of the people in the world were living in a water shortage area. To meet the needs of people in all aspects of water resources, the UN has started to address the global water crisis caused by water scarcity and pollution. The UN released the International Decade for Action on Water for Sustainable Development, 2018–2028, to promote sustainable development and comprehensive management of water resources [2]. Ranking only second to food security, water security is the basis on which to guarantee the health of humanity and a sustainable economy, society and ecological environment [3]. Some scholars believe that economic development relies largely on water supply and that the unsustainable development of water resources could greatly hinder progress of society [4]. It is necessary to carry out a scientific sustainability assessment of water resources to promote their sustainable development, which is of great significance when searching for measures to realize the sustainable use of water resources and the sustainable development of society, economy and ecology.

The International Conference on Water and Environment held in 1992 first illustrated the role water resources played in the environment and the development of society. The conference explicitly raised the issue of the water resources system and sustainability research [5]. Since then, more and more scholars at home and abroad have started to implement research on the sustainability of water resource use. Based on a literature review of sustainability assessment, Zijp et al. [6] put forward options and tools for selecting sustainability assessment methods, testing them in a case study and making strategic decisions for sustainable resource recovery of wastewater in the Netherlands. Chaves and Alipaz [7] came up with watershed sustainability indicators—WSI. These indicators comprehensively take the elements of hydrology, environment, human livelihood, policy, and so on, into consideration and select relevant indicators to assess the present status of watershed management thoroughly for a certain watershed in a specific period. Gleick [8] constructs a set of urban sustainable development indicators from various urban aspects to provide long-term planning and recommendations for urban water management.

A key step to assess the sustainability of water resource use is constructing a reasonable assessment indicator system. Sustainable development is the organic integration of ecosystem sustainability, economic sustainability and social sustainability, pursuing harmony within humans and between humans and nature. In constructing the ISSAWRU, scholars consider social, economic and natural environmental factors comprehensively, while differing in specific criteria. Mojtaba et al. [9] developed a Sustainability Assessment framework comprising technical, environmental, economic and social issues to assess sustainable water management in the Mashhad Basin. Huang et al. [10] discuss the ISSAWRU on the basis of a generalized classification of water resources assessment. In addition, many scholars have established an ISSAWRU by using the DPSIR model (Driving Force-Pressure-State-Impact-Response), which is a conceptual model for solving problems of environmental resources with a criterion layer of five causally related factors: driver, pressure, state, impact and response [11,12,13,14]. Apart from DPSIR, water footprint theory has also been used to construct ISSAWRU [15,16,17,18].

Selecting appropriate methods is a vital step in assessment and decision-making. As of now, the sustainability of water resources use has been assessed by various methods, such as the objective method, subjective method, combined method, etc. Methods being used frequently are Analytic Hierarchy Process, fuzzy comprehensive assessment method, method of matter element analysis, TOPSIS (Technique for Order Preference by Similarity to An Ideal Solution), Grey Correlation Analysis, etc. [19,20,21,22,23]. Different methods can make a difference in assessment results. For instance, Analytic Hierarchy Process and the fuzzy comprehensive assessment method focus on the judgment of experts which is based on personal bias, and the experience of experts often varies greatly. Matter element analysis is suitable for multi-factor assessment, but indicator incompatibility has not been fully resolved. TOPSIS and Grey Correlation Analysis are suitable for comparing multiple options. The TOPSIS method lacks stability due to its own limitations, which can cause changes in positive and negative ideal solutions [24]. Grey Correlation Analysis has a limited scope of application and enquires sample data with characteristics of time series. The sustainability assessment of water resources use is highly comprehensive, involving water resource conditions, development and utilization of water resources, development of socio-economy, eco-environment, etc. Its assessment indicators and grading standard for these indicators are random and fuzzy. Cloud model is a mathematical model based on the normal distribution and bell-shaped subordinate function, which is used to realize the uncertainty transformation between qualitative and quantitative aspects of a phenomenon and things in the objective world and can effectively solve the problem of fuzziness, randomness and dispersion in the process of water resources evaluation, with pervasive characteristics. [25]. At present, there is an extensive literature associated with the cloud model in terms of water resources assessment, forecasting and reporting, option selection and risk assessment [24,25,26,27,28,29], which indicate the feasibility and adaptability in the application to hydrology of the cloud model.

The studies above make crucial contributions to the sustainability assessment of water resources use, lay a foundation and point out the direction for future research. So far, most of the studies on WRSU have been based on city or province level [30,31,32], with few studies researching the overall level of China and presenting spatial characteristics [33]. Therefore, this paper will take 31 provinces in China as the research object. The research contains four aspects. First, according to the construction principle of the assessment system and existing literature, ISSAWRU is constructed from three aspects: water resources condition, socio-economy and ecological environment. Second, the entropy weight method and cloud model theory are combined to construct a water resources sustainability assessment model. Third, the ISSAWRU and water resources sustainability assessment model are used to evaluate the sustainability of water resources use in 31 provinces and cities in China in 2019, and reliability was tested by comparing the results with those obtained using the TOPSIS method. Fourth, the sustainability of water resource use in China is analyzed deeply in terms of spatial characteristics. The research contributions of this paper are as follows. First, the research method combines the entropy weight method and cloud model to solve the problem of randomness and fuzziness in the evaluation of sustainable water resources use, making the evaluation results more realistic and objective. Second, the research results enrich and improve the indicator assessment system for sustainable water resources use in China and extend the application scope of the cloud model. Third, this paper conducts an assessment of the sustainable use of water resources at the overall level of China, analyses these from the point of view of spatial characteristics, and provides ideas and references for promoting sustainable water resources use in China, further removing barriers to regional economic development and achieving balanced development.

## 2. Study Area and Methods

### 2.1. Study Area

China is located in eastern Asia and on the west coast of the Pacific Ocean (3°51′–53°33′ N; 73°33′–135°05′ E). It is a vast country that spans a wide range of latitudes and has a wide variety of climates. In terms of climate type, the east has a monsoon climate, the northwest has a temperate continental climate and the Qinghai-Tibet Plateau has an alpine climate. Precipitation decreases from the southeastern coast to the northwestern interior, and the uneven regional distribution of precipitation has resulted in an imbalance in soil and water resources across the country. The annual and interannual variations of precipitation and runoff are great. The greatest concentration of precipitation is in the pre-hill areas of the Yellow Huaihai Plain, where floods are often in the form of heavy rainfall, and in some years the one-day heavy rainfall exceeds the multi-year average annual precipitation. In some years, there are droughts in the north and floods in the south, while in other years there are floods in the north and droughts in the south. The above characteristics of water resources are the main reasons for the frequency of floods and droughts and the instability of agricultural production in China [34,35].

In 2020, the total freshwater resources in China were 3.16 trillion cubic meters, accounting for 6% of global water resources, while the total Chinese population was 1.4 billion, accounting for 18% of the world’s population [34,35]. Although China ranks high in the world in terms of total water resources, its per capita water resources are only about 2254.7 m^3^, less than one-half of the world average, and it is one of the “water-vulnerable countries” as defined by the United Nations Environment Programme [34,35]. In 2019, the area of soil erosion in the country was 2,710,800 square kilometers, and the area of hydraulic erosion reached 1,134,700 square kilometers [36]. Massive soil erosion had reduced cultivated land and expanded the area of desertification, leading to continued deterioration of ecological environment and further siltation of rivers and lakes, thus exacerbating natural disasters such as floods, droughts and sandstorms. In 2020, direct economic losses caused by floods were 266.98-billion-yuan, accounting for 0.26% of GDP in the current year, and several regions of the country were affected by drought, causing problems in drinking water for millions of urban and rural people as well as large amounts of livestock, with direct economic losses of 18.6 billion yuan in total [37]. In the same year, the proportion of water used in agriculture in China was as high as 62.14% of total water consumption, and the effective utilization coefficient of irrigation water was only 0.565 [38]. Due to the unreasonable distribution of water resources in all fields and regions of agriculture as well as the low efficiency of irrigation, there was a substantial waste of water in agriculture, with only half of the water resources effectively serving the purpose of irrigation. Recently, with the dramatic expansion of cities and the rapid development of industry, water pollution has become severer. Affected by the monsoon climate, the spatial and seasonal distribution of water resources in China has been extremely unbalanced, which has further hindered the rational development and utilization of water resources [39]. In general, China is currently facing water shortages, serious soil erosion, frequent floods and droughts, serious waste of water resources, aggravated water pollution and uneven distribution of water resources, which influence people’s living conditions and economic development, damages the ecological environment and greatly restricts the sustainable development of water resources. This paper took 31 provinces in China as research objects, established China’s sustainable water resources use evaluation index system and sustainable water resources use evaluation model and systematically measured and analyzed China’s water resources sustainable use level, in order to explore and improve the sustainability of water resource use. 

### 2.2. Construction of an Indicators System

Based on consulting a large number of relevant literature, following the principles of systematic, typical, comprehensive, scientific and comparable construction of an evaluation index system, combined with China ‘s natural geographical environment and human characteristics, this paper constructed the ISSAWRU, which included three subsystems, water resources conditions, socio-economy and eco-environment, and 18 secondary assessment indicators. The water resources condition subsystem mainly reflected the natural conditions and development and utilization of water resources, the socio-economic subsystem mainly contains the aspects of supply, demand and allocation of water resources, and the eco-environmental subsystem concentrates more on the level of quality management of water resources. Additionally, this paper referred to relevant document standards and literature [25,26,27,28,29,39,40,41] and experts’ conclusions, investigated the actual situation of water resources in China and determined the five-level grading standard for assessment indicators. The higher the class, the stronger the sustainability of water resource use. The grading standards of specific assessment indicators are shown in Table 1.

### 2.3. Data Source

This paper selected the data of 31 provinces in China in 2019 as a research sample (due to the availability of data, Hong Kong, Macau and Taiwan are excluded). The original data were mainly from the 2020 Statistical Yearbook on Environment, and a smaller amount of data were taken from the 2019 China Water Resources Bulletin, 2020 China Statistical Yearbook, 2019 China Soil and Water Conservation Bulletin, 2021 China Statistical Yearbook and the official website—Ministry of Civil Affairs of the People’s Republic of China, which formed an original data set through systematic collecting and sorting. All data were publicly available online.

### 2.4. The Entropy Method for Determining the Weight of Each Indicator

The weight of assessment indicators was determined by the entropy weight method, which was an objective assignment method and relied on the discreteness of the data itself. The greater the discreteness of the assessment indicators, the greater the weight [42].

(1) Based on the initial data set of *n* evaluation indices of *m* schemes, the eigenvalue matrix is established.

(1) $$X={\left(x_{ij}\right)}_{m\times n}(i=1,2,\ldots ,m;j=1,2,\ldots ,n)$$

(2) Standardize the matrix due to the differences in the dimension and order of magnitude of each indicator. The processing equations for positive and negative indicators are as follows.

(2) $$z_{ij}=\frac{x_{ij}-x_{j}^{\min}}{x_{j}^{\max}-x_{j}^{\min}}$$

(3) $$z_{ij}=\frac{x_{j}^{\max}-x_{ij}}{x_{j}^{\max}-x_{j}^{\min}}$$

Obtain standardized matrix $Z={\left(z_{ij}\right)}_{m\times n}(i=1,2,\ldots ,m;j=1,2,\ldots ,n)$, *x_j_*^min^, *x_j_*^max^ are the minimum and maximum values of the same indicator *x_j_* in different scenarios, respectively.

(3) Define the entropy of the indicators according to the traditional concept of entropy.

(4) $$h_{j}=-\frac{\sum_{i=1}^{m}f_{ij}\ln f_{ij}}{\ln m}$$

In the Equation above, $f_{ij}=\frac{z_{ij}}{\sum_{i=1}^{m}z_{ij}}$.

To eliminate the influence of the entropy value on the calculation result, when $z_{ij}=0$, make $z_{ij}$ a translation correction and let ${z^{\prime }}_{ij}=z_{ij}+0.01$.

(4) The entropy weight of the j-th evaluation indicator:

(5) $$w_{j}=\frac{g_{j}}{\sum_{j=1}^{n}g_{j}}$$

In the Equation above, $g_{j}=1-h_{j}$.

### 2.5. Assessment Methodology Based on Cloud Model

**Figure 1:**
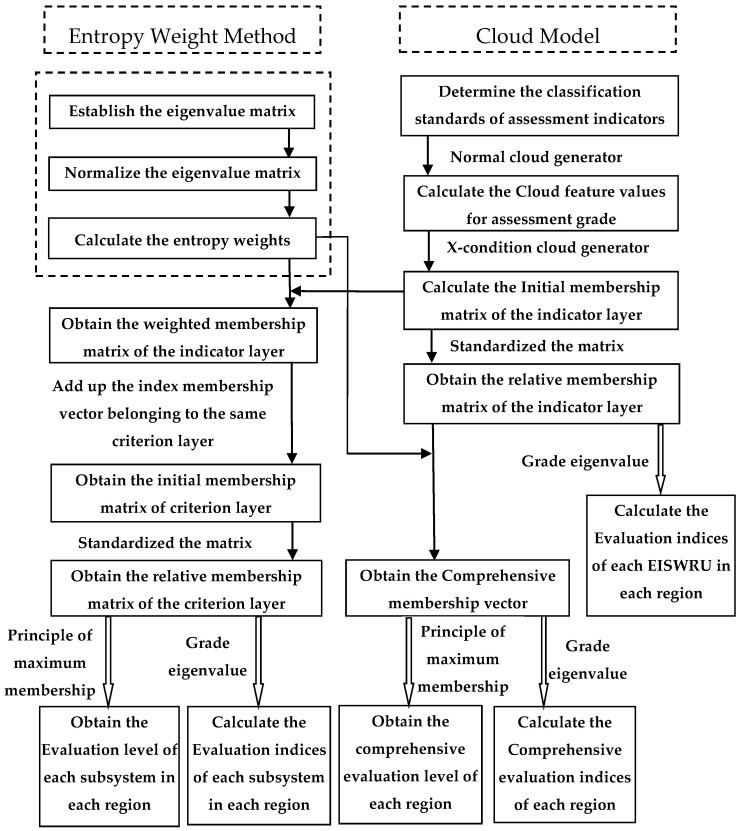
The flow diagram of the Entropy-Cloud model.

This paper constructed an assessment model for the sustainability assessment of water resources use in China based on the cloud model, a mathematical model based on normal distribution and bell-shaped affiliation function, which is used to realize the uncertainty transformation between qualitative and quantitative aspects of a phenomenon and objects in the objective world, which can effectively solve the problem of fuzziness, randomness and dispersion in the process of sustainability assessment of water resources. The cloud model is widely used in water resources evaluation because of its universal applicability [43]. The flow diagram of the entropy-cloud model is shown in Figure 1.

#### 2.5.1. Determine the Normal Cloud Criteria for Each Indicator

Establish a set of assessment grades $C=\{C1,C2,\cdots ,C_{k},\cdots ,Cp\}$(k=1,2,⋯,p), generally, take *p* = 4 or *p* = 5 (in this paper *p* = 5). Divide the effective theoretical domain of each evaluation indicator *a_j_* into *p* subintervals according to the assessment indicator grading criteria. The *k*-th subinterval is $\left[C_{jk}^{\min},C_{jk}^{\max}\right]$ and cloud feature values corresponding to each subinterval $\left(E_{x},E_{n},H_{e}\right)$ can be expressed in Equation (6). The forward normal cloud generator can be used to generate a standard cloud of assessment ratings for each assessment indicator.

(6) $$\begin{array}{l} E_{x}=(C_{jk}^{\max}+C_{jk}^{\min})/2\\ E_{n}=(C_{jk}^{\max}-C_{jk}^{\min})/2.355\\ H_{e}=u \end{array}$$

Among the equation, *u* is a constant and can be adjusted as required during the test. For intervals with only one side boundary, the endpoint values are filled according to the actual data.

#### 2.5.2. Calculate the Degree of Membership

By using the X-conditional cloud generator, calculate the membership degree of assessment indicator *a_j_* belonging to assessment grade *C_k_*. The average membership degree $y_{jk}=\frac{1}{v}\sum_{l=1}^{v}y_{jlk}$ is obtained by averaging $y_{jlk}$ in the generated *v* (In this study, *v* = 1000) cloud droplets $\left(x_{jlk},y_{jlk})(j=1,2,\cdots ,n;l=1,2,\cdots ,v;k=1,2,\cdots ,p\right)$. The average membership degree of indicator *a_j_* at *p* assessment grade constitutes the index initial membership vector $y_{j}=[y_{j1},y_{j2},\ldots ,y_{jp}]$. Initial membership vectors of n indexes form initial membership matrix of indicator layer $R_{1}={\left(y_{jk}\right)}_{n\times p}$. The initial membership vector of each assessment indicator is multiplied by the weight of the corresponding assessment indicator to obtain the membership vector of each evaluation indicator. Then, the index membership vector belonging to the same criterion layer is added to obtain the initial membership vector of each criterion layer (assuming that there are *s* criterion layers, *s* = 3 in this study). Initial membership vector of *s* criterion layer forms initial membership matrix of criterion layer $R_{2}={\left(r_{fk}\right)}_{s\times p}$(f=1,2,⋯,s).

The relative membership matrix of indicator layer and criterion layer $R_{3}={\left({y^{\prime }}_{jk}\right)}_{n\times p},R_{4}=({r^{\prime }}_{fk}{)}_{s\times p}$ is calculated by Equations (7) and (8), respectively:

(7) $${y^{\prime }}_{jk}=\frac{y_{jk}}{\sum_{k=1}^{p}y_{jk}}$$

(8) $${r^{\prime }}_{fk}=\frac{r_{fk}}{\sum_{k=1}^{p}r_{fk}}$$

Calculation of comprehensive membership vector *D* by Equation (9):

(9) $$D=WR_{3}={\left(d_{k}\right)}_{1\times p}$$

among which $W={\left[w_{1},w_{2},\cdots ,w_{n}\right]}^{T}$ is the index weight vector based on the entropy weight method, and *d_k_* is the membership degree of the measured object belonging to each level.

#### 2.5.3. Determine the Final Results of the Assessment

According to the maximum membership principle, the results of the comprehensive assessment grade of each region can be determined by the comprehensive membership vector. Assessment results of each subsystem in each region are determined by the relative membership matrix of the criterion layer. To facilitate comparisons between regions, assign grade eigenvalues $Z=\{z_{1},z_{2}\ldots z_{p}\}$ to each level in the assessment level set *C*. Adopting the weighted average method, use Equations (10)–(12) to calculate the assessment index of each indicator *O_j_*, the assessment index of each criterion level *T_f_* and the comprehensive evaluation index of the measured object *Q*, respectively.

(10) $$O_{j}=\sum_{k=1}^{p}z_{k}{y^{\prime }}_{jk}$$

(11) $$T_{f}=\sum_{k=1}^{p}z_{k}{r^{\prime }}_{fk}$$

(12) $$Q=\sum_{k=1}^{p}z_{k}d_{k}$$

## 3. Results and Discussion

### 3.1. Find the Weight of Assessment Indicators through Entropy Weighting Method

This paper constructed ISSAWRU with three criterion layers and 18 indicator layers. At the beginning of the evaluation, corresponding weights were assigned to each assessment indicator. This paper adopted the entropy weight method to calculate the weight of each evaluation index. First, the original data was normalized using Equations (2) and (3) to obtain a normalized matrix. Then, Equations (4) and (5) were used to process the normalized matrix in turn to obtain the weight of the evaluation index. Table 2 shows the results. As can be seen from Table 2, each assessment indicator had a different degree of influence on the sustainability of water resource use. The weight of the water resources condition subsystem in the criterion layer reaches 0.465, indicating that the water resources condition subsystem had the greatest influence on the sustainability of water resources use, in which the weight of the water resources per capita is 0.306—the greatest weight among all indicators. The importance of the socio-economic and eco-environmental subsystems showed little difference, with weights of 0.291 and 0.244, respectively. The assessment indicator with the greatest weight in the socio-economic subsystem was the irrigation rate of cultivated land (0.066) and the assessment indicator with the greatest weight in the eco-environmental subsystem was the eco-environmental water use rate (0.136). 

### 3.2. Cloud Feature Values for Assessment Grades

This paper simulated the evaluation grade standard cloud with a forward cloud generator and adjusted the parameters to find the appropriate dispersion, so as to determine the appropriate cloud characteristic value. Based on the grading criteria of EISWRU in China in Table 1, the endpoint values of the actual data were taken as the boundary values to obtain the corresponding assessment intervals and the cloud characteristic values Ex and En are calculated according to Equation (6). Python was used to write the code for the forward cloud generator, the code is run, and the parameter u was adjusted according to the dispersion of the cloud map, resulting in the cloud characteristic values for each assessment indicator corresponding to each assessment grade, the results of which are shown in Table 3.

### 3.3. Assessment Results

After the cloud eigenvalues were obtained, the evaluation indices of each evaluation index of the sustainable water resource use, the assessment grade results and evaluation indices of each subsystem and the comprehensive assessment grade results and comprehensive evaluation indices of 31 provinces of China were calculated. The initial data set and cloud eigenvalues (Table 3) were substituted into the X-condition cloud generator to obtain the initial membership matrix of the indicator layer of 31 provinces. Combined with Equation (7), the initial membership matrix of the indicator layer was standardized to obtain the relative membership matrix of the indicator layer. The initial membership vector of all indexes was multiplied by the weight of corresponding indices to obtain the membership vector of indexes. Then, the index membership vector belonging to the same criterion layer was added to obtain the initial membership vector of each criterion layer. All initial membership vectors of the criterion layer constituted initial membership matrix of the criterion layer. Then, Equation (8) was standardized to obtain the relative membership matrix of the criterion layer. According to the principle of maximum membership, the evaluation results of each subsystem in each region were determined, as shown in Table 4. Using Equation (9), the relative membership matrix of the indicator layer was multiplied by weight vector to obtain a comprehensive membership vector. According to the principle of maximum membership, the comprehensive evaluation level of each region was determined, as shown in Table 4.

In order to facilitate comparisons between regions and to analyze the evaluation results more deeply, an evaluation grade was assigned to the grade eigenvalue to calculate the evaluation index (1, 2, 3, 4, and 5 are characteristic values of grades I, II, III, IV, V). The higher the grade, the greater the grade eigenvalues. Using Equation (10) and the characteristic value of the evaluation grade, the evaluation indices of each EISWRU in various provinces in China were obtained. The evaluation indices of water resources condition subsystem, socio-economic subsystem and eco-environmental subsystem are obtained by using Equation (11) and evaluation grade eigenvalue. The specific indices and rankings are shown in Table 5. The comprehensive evaluation indices in various provinces in China were obtained by using Equation (12) and the characteristic value of evaluation grade. The specific indices and rankings are shown in Table 5. The calculated evaluation index value interval was [1,5]. The larger the evaluation index value, the stronger the sustainability of water resources use in the region, and vice versa.

### 3.4. Comparison of the Results of Assessment

To verify the accuracy of the cloud model, this paper standardized the original data, obtained the weights by weight method and used TOPSIS to assess the sustainable use of water resources in China [24,29]. 

Table 4 demonstrates that the evaluation results of other regions are generally consistent, except that those of the two provinces of Tibet and Qinghai are quite different. It may be that the water resources per capita and the household wastewater emissions in urban areas in Tibet and Qinghai are too different from other regions. There is a large difference between Tibet and other regions in terms of development and utilization rate of water resources and the COD emissions in wastewater. Moreover, since the weight of water resources per capita in the evaluation process accounts for a large proportion of the weight, there is distortion of the evaluation criteria of the evaluation indices under the computer system of TOPSIS. Therefore, the evaluation levels of Tibet and Qinghai present completely different results. In the evaluation of cloud models, the evaluation criteria are equated according to the overall level and would not be affected by a certain area with an unusual level, so the evaluation results of the cloud model are more reliable.

### 3.5. Analysis of Evaluation Results

#### 3.5.1. Analysis of Comprehensive Evaluation Results of Sustainability of Water Resource Use

**Figure 2:**
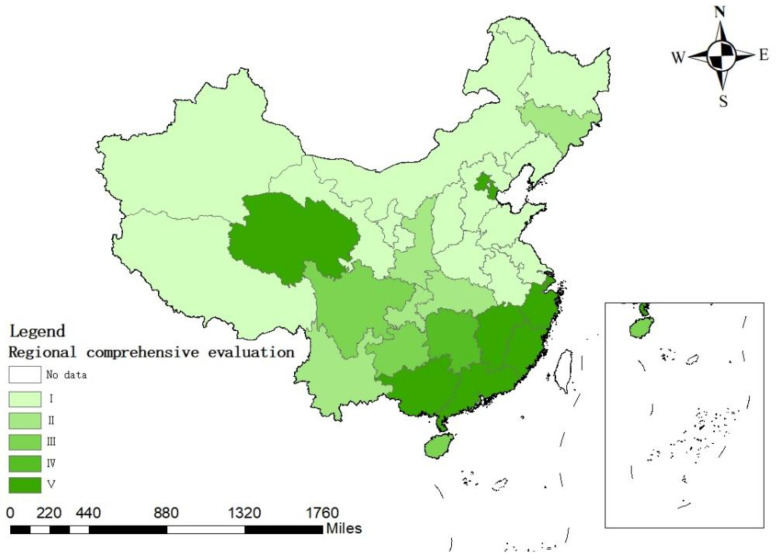
Regional comprehensive evaluation results of the sustainability of water resources use in China.

From Table 5 Regional comprehensive evaluation indices, the evaluation indices of 31 provinces are all within the range of [2,4], and the overall distribution is relatively concentrated without extreme values. From the evaluation results of the cloud model, the higher the evaluation level, the higher the sustainability of water resource use. From Table 4, it can be seen that there are 13 regions with a Class I comprehensive evaluation level in sustainability of water resource use, accounting for 41.94%. There are five regions with Class II evaluation level, accounting for 16.13%. There are three regions with Class III evaluation level, accounting for 9.68%. There is one area with an evaluation level of Class IV, accounting for 3.23%. There are nine areas with an evaluation level of Class V, accounting for 29.03%. Figure 2 shows the spatial distribution of the evaluation grades of sustainable water resources use in various provinces in China. It can be seen that the areas with high levels of sustainability of water resource use are mainly concentrated in the southeast, while the levels of sustainability of water resource use in the western and northern regions are extremely low. The sustainability level of water resources in most areas is at the lowest level, and further analysis of the reasons and corresponding countermeasures are required.

In terms of comprehensive evaluation results, the grades of sustainability of water resource use in Beijing, Tianjin and Shanghai are the highest, while their grades for water resource condition subsystem are the lowest. The water resource condition subsystems of Heilongjiang, Tibet and Xinjiang were at a high grade; however, their grades of sustainability of water resource use are all at the lowest grade. This shows that economic development is conducive to the increase of water resource use efficiency, while the development of economically backward regions is often more dependent on water resources, whose unreasonable industrial structure and methods of use can lead to greater pressure on water resource use and can hardly provide sufficient funds to guarantee the supply and protection of water resources. The assessment of socio-economic subsystems in Shanghai, Guangdong and Chongqing are at a high grade, but the grade of their eco-environment subsystem is the lowest. This shows that the prosperity of the economy and the expanding population size has led to a dramatic upward trend in water demand and the continuous improvement of water use efficiency. Water consumption in the urban area can easily exceed the carrying capacity of the local water resources and the water environment when a region develops to a certain stage. Therefore, blindly increasing the water resources development rate not only aggravates the imbalance between the water resources supply and demand, but generates high pollution pressure on the water environment, leading to eco-imbalances and unsustainability of water resources use.

#### 3.5.2. Analysis of Assessment Results of Water Resource Condition Subsystem

From Table 4, it can be seen that there are 12 regions with a Class I evaluation level of water resource condition subsystem, accounting for 38.715%. There are five regions with the evaluation level of Class II, accounting for 16.13%. There are three regions with a Class III evaluation level accounting for 9.68%. There are two regions with an evaluation level of Class IV, accounting for 6.45%, and eight regions with an evaluation level of Class V, accounting for 25.81%. 

**Figure 3:**
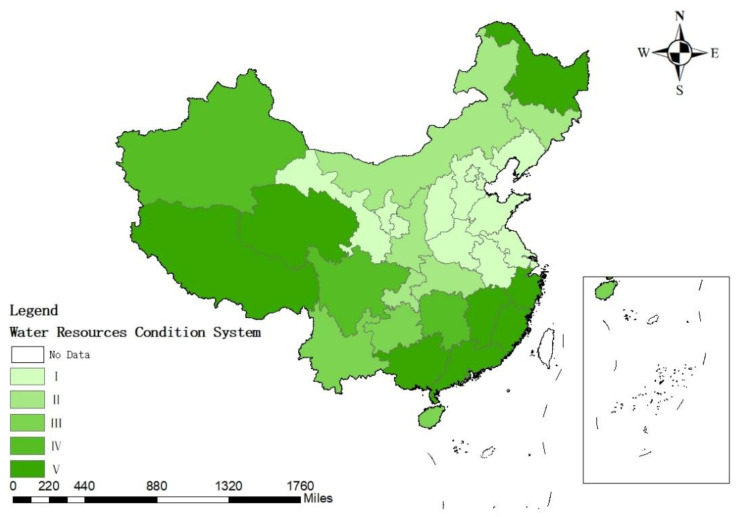
Assessment results of China’s water resource condition subsystem.

Combining Table 5 with Figure 3’s assessment results of China’s water resource condition subsystem, most of the Chinese regions’ water resources conditions have a high evaluation level and the specific evaluation index is located in [2, 4.9], with obvious regional differences. The southeastern region has the best water resource conditions, followed by the western region and Heilongjiang Province, while the eastern region, Gansu Province and Ningxia Hui Autonomous Region have the worst water resource conditions. 

Further analysis shows that Guangxi, Guangdong, Jiangxi, Fujian and Zhejiang located in the southeast have high annual precipitation, per capita water resources, water resources development and utilization rate and water production modulus evaluation index; that is, the water resources endowment of these five provinces is very high and the amount of water is highly sufficient and can fully meet needs for production and for the life of the local people. Tibet, Xinjiang, Qinghai in the west and Heilongjiang in the north have the same characteristics of low annual precipitation and low water production modulus. However, the amount of water per capita is extremely large and the development and utilization rate of water resources is low. The water resources per capita is the most important indicator in the assessment indicator system, so these four provinces have higher evaluation levels. Fundamentally, these provinces have poor water resource endowments, but they are vast and sparsely populated and the amount of water is sufficient for the needs of production and for the life of local residents. The precipitation and water production modulus of Shanghai are high, but the population density of Shanghai is also extremely high, the economy is developed, there is a large demand for water resources and the development and utilization of water resources is very high, so the evaluation of water resources condition of Shanghai is not high. The five regions, Beijing, Tianjin, Hebei, Jiangsu in the east and Ningxia in the middle, have poor water resource endowments and high water resource development and utilization rates. Due to their densely populated and developed economy, local demand for water can be too large to be met merely by their own water resources. In addition, the extremely high water resource development and utilization rate can cause environmental imbalance, affecting the sustainable use of water resources. The six regions of Shanxi, Shandong, Henan, Anhui, Liaoning and Gansu have poor water resources endowment and the per capita water resource are relatively small, but the degree of water resources development and utilization is relatively low. The potential of water resources can be tapped appropriately to meet people’s demand for water.

#### 3.5.3. Analysis of Assessment Results Socio-Economic Subsystem

From Table 4, it is clear that there are 10 regions with the evaluation level of the socio-economic subsystem at Class I, accounting for 32.26%. There are 10 regions with the evaluation level at Class II, accounting for 32.26%. There are seven regions with the evaluation level at Class III, accounting for 22.58%. There are two regions with the evaluation level at Class IV, accounting for 6.45% and there are two regions with an evaluation level of Class V, accounting for 6.45%. 

**Figure 4:**
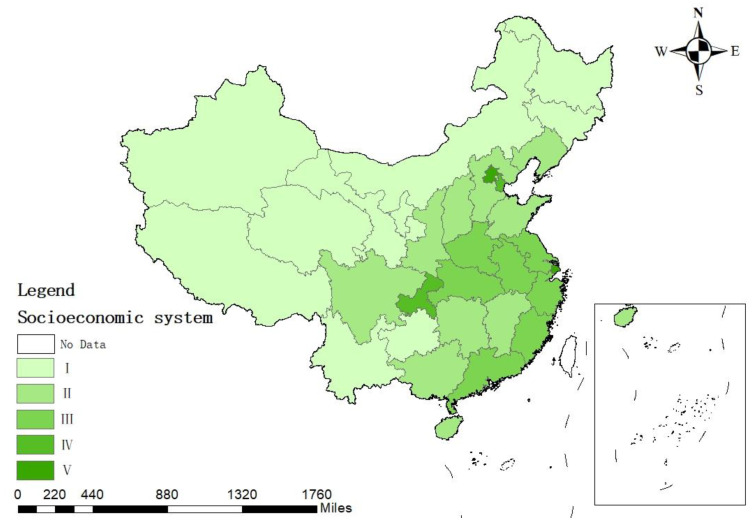
Assessment results of China’s socio-economic subsystem.

Combined with Table 5 and Figure 4’s assessment results for China’s socio-economic subsystem, it is clear that, in the sustainability assessment of water resource use in China, the evaluation level of socio-economic subsystems in most regions is low and there is a clear spatial trend. The evaluation level of the southeast is generally higher than that of the west and north. In the evaluation of socio-economic subsystems, the proportion of industrial water use, agricultural water use, household water use, irrigation rate of cultivated land and effective utilization coefficient of cultivated land irrigation water have a larger weight and show the greatest influence on evaluation results. 

Shanghai and Beijing are at the highest evaluation level and the evaluation index is above 4. The proportion of agricultural water use in these two cities is relatively low and the irrigation rate of cultivated land is relatively high. However, in terms of the effective utilization coefficient of cultivated land irrigation water, Beijing has a high evaluation, while Shanghai is slightly less efficient. Beijing has a low proportion of industrial water use and a high proportion of household water use, while Shanghai is just the opposite. In the evaluation of socio-economic subsystems, Chongqing has a high evaluation level and performs well in terms of water use structure and economy, but the evaluation of cultivated land irrigation rate and effective utilization coefficient of cultivated land irrigation water is low and it is of necessity to strengthen water saving in irrigation areas. Except for Shanghai, Beijing, Tianjin and Chongqing, the water use structure evaluation of other regions is low. Inner Mongolia, Jilin, Heilongjiang, Guizhou, Yunnan, Tibet, Gansu, Qinghai, Ningxia and Xinjiang are ranked at the lowest level in the socio-economic subsystem, with low water use structure and agricultural water use efficiency, among which, the water economy of Heilongjiang, Tibet and Xinjiang are relatively poor. The per capita water resources of Tibet and Xinjiang are relatively sufficient, but their per capita water consumption is also far higher than that of other regions. It is necessary to promote a water-saving culture, enhance the public’s water-saving awareness and decrease the waste of water resources.

#### 3.5.4. Analysis of Assessment Results of Eco-Environmental Subsystem

From Table 4, it can be seen that there are 15 regions with the evaluation level of the eco-environmental subsystem at Class I, accounting for 48.39%. There are eight regions with an evaluation level of Class II, accounting for 25.81%. There are two regions with the evaluation level of Class III, accounting for 6.45%. There are two regions with the evaluation level Class IV, accounting for 6.45% and there are four regions with an evaluation level of Class V, accounting for 12.9%. 

**Figure 5:**
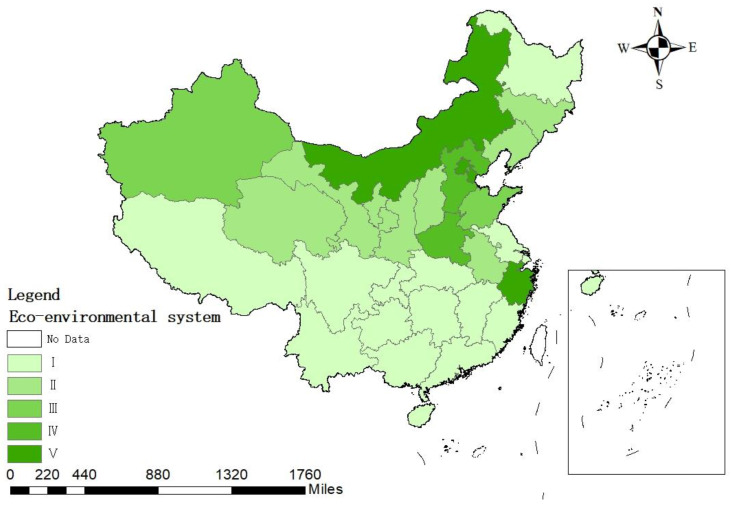
Assessment results of China’s eco-environmental subsystem.

Combined with Table 5 and Figure 5’s assessment results of eco-environmental subsystem, it is clear that, in the sustainability assessment of water resource use in China, the evaluation level of eco-environmental subsystems in most regions is low and there is a clear spatial trend. The evaluation level of the northern region is generally higher and the evaluation index of each region is distributed in [1.9, 4.7], with obvious regional differences. In the evaluation of the eco-environmental subsystem, the eco-environmental water use rate has the largest weight and has the most significant impact on the evaluation results. The continuous increase of national economic water consumption can crowd out eco-environmental water use, resulting in a further shortage of local water resources, degradation of natural vegetation and aggravation of desertification, seriously affecting the sustainability of water resource use. 

Beijing has the highest eco-environmental evaluation level, with an evaluation index of 4.634, far ahead of other regions. The second-ranked Tianjin evaluation index is 4.012, but its forest coverage rate and sewage treatment rate are relatively low. Except for Beijing and Tianjin, only the six provinces of Henan, Hebei, Inner Mongolia, Shandong, Zhejiang and Jilin have an evaluation index above 3; 74% of the provinces in the country are at a relatively low level in the evaluation of the eco-environmental subsystem, and the eco-environmental water use rate and sewage treatment rate in most areas are relatively low. It is necessary to adjust the water use structure and strengthen pollution control. Shanxi, Inner Mongolia, Gansu, and Xinjiang have low forest coverage and serious soil erosion. Investment in environmental protection and frequency of tree planting should be increased. The wastewater in Jiangsu and Guangdong contains a very large amount of COD emissions, and the sewage treatment rate is also low, which aggravates the pollution of the water environment and seriously affects the safety of water resources.

## 4. Conclusions

Based on the relationship Human–Resource–Nature, this paper established an ISSAWRU in China from three perspectives: water resources conditions, socio-economy and ecological environment and constructed the assessment model by using the entropy weight method and the cloud model. The ISSAWRU and assessment model was used for assessing and analyzing the sustainability of water resources use in 31 provinces in China in 2019. The main findings are as follows.

(1) The indicator system constructed in this paper included three aspects: water resources condition subsystem, socio-economic subsystem and eco-environment subsystem, with weights of 0.465, 0.291 and 0.244, respectively. The influence on the sustainability of water resources use was in the following order: water resources condition subsystem > socio-economic subsystem > eco-environment subsystem. Among the EISWRU, the water resources per capita accounted for the largest proportion, and water consumption structure, agricultural water use efficiency and forest coverage present a greater influence on the sustainability assessment of water resources use.
(2) The overall degree of sustainability of water resource use in China’s 31 provinces is not high, 42% of the regions have unsustainable water resources use and there is a clear spatial distribution trend, with the southeastern regions and economically developed regions having a higher degree of sustainability of water resource use. In terms of the water resources condition subsystem, the western parts with lower population density, Heilongjiang Province and the southern regions with abundant water resources have better water resources conditions. In terms of the socio-economic subsystem, the southeast coastal regions are rated higher than the western and northern regions, with the economically developed regions of Beijing, Tianjin, Shanghai and Chongqing rated the highest. In terms of the eco-environment subsystem, the ecological environment subsystem in the southern region is at the lowest rating and the northern regions are normally rated higher than the southern regions, except for Zhejiang. The irrational industrial structure of less economically developed regions often results in a poor water consumption structure, leading to unsustainable water resource use. Water resources in economically developed regions are used in a more efficient way, but excessive exploitation could exceed the carrying capacity of the water environment and easily cause environmental pollution and ecological imbalance.
(3) Each of China’s 31 provinces has a different level of sustainability in the use of water resources and faces different challenges. Each region should develop measures to ensure water security according to its local conditions. For example, the six regions of Shanxi, Shandong, Henan, Anhui, Liaoning and Gansu are poorly endowed with water resources and have low water resources per capita, but they are in a low level of water resources development and utilization, so they could achieve their water resources potential and strengthen the use of non-conventional water resources.

This paper assessed the sustainability of water resources use in China, not only for 31 provinces in a comprehensive manner, but also for three sub-systems and 18 assessment indicators individually, in order to provide a reference for decision makers to trace the root of influencing factors and achieve water sustainability. This study used the entropy weight method and cloud model to construct an evaluation model for sustainability of water resources use in China, which considered both fuzziness and randomness and greatly avoided subjectivity in the assessment process. The research results enrich and improve the evaluation index system of sustainable water resources use in China, expanding the application scope of the cloud model. This paper conducts an assessment of the sustainable use of water resources at the overall level of China, analyses from the spatial characteristics, and provides ideas and references for promoting sustainable water resource use, further removing obstacles to regional economic development and realizing balanced development. It should be noted that the assessment of the sustainability of water resources use involved many aspects: society and economy, natural environment, etc., and, influenced by the availability of data, the assessment indicators selected in this paper may not be comprehensive and perfect. As more information becomes available and abundant, future research will further enrich the ISSAWRU in China and incorporate dynamic simulations to study the sustainability of water resources in cities in a more in-depth and comprehensive manner.

## Figures and Tables

**Table 1:** The classification standards for sustainability assessment of water resources use.

Criterion Layer	Indicator Layer	Attribute	Class I	Class II	Class III	Class IV	Class V
Water resource condition Subsystem A1	Annual precipitation (a1) (mm)	Positive	<500	500~900	900~1250	1250~1600	>1600
	Water resources per capita (a2) (m^3^)	Positive	<1040	1040~2030	2030~3020	3020~4010	>4010
	Water resources development and utilization rate (a3) (%)	Negative	>2.4	2.4~1.8	1.8~1.2	1.2~0.6	<0.6
	Water production modulus (a4) (10^4^ m^3^/10^6^ m^2^)	Positive	<28	28~54	54~80	80~106	>106
Socio-economic subsystem A2	Industrial water consumption rate (a5) (%)	Positive	<0.13	0.13~0.25	0.25~0.35	0.35~0.47	>0.47
	Agricultural water consumption rate (a6) (%)	Negative	>0.72	0.72~0.57	0.57~0.4	0.4~0.25	<0.25
	Household water consumption rate (a7) (%)	Positive	<0.11	0.11~0.2	0.2~0.28	0.28~0.36	>0.36
	Household wastewater emissions in urban area (a8) (10^4^ m^3^)	Negative	>361,900	361,900~273800	273,800~185,800	185,800~97,700	<97,700
	Water consumption per capita (a9) (m^3^)	Negative	>840	840~700	700~500	500~350	<350
	Water consumption per 10,000 yuan of GDP (a10) (m^3^/10,000yuan)	Negative	>350	350~260	260~180	180~95	<95
	Water consumption per 10,000 yuan of industrial added value (a11) (m^3^)	Negative	>90	90~70	70~50	50~30	<30
	Cultivated land irrigation rate of (a12) (%)	Positive	<0.44	0.44~0.62	0.62~0.81	0.81~1	>1
	Effective utilization coefficient of cultivated land irrigation water (a13)	Positive	<0.5	0.5~0.57	0.57~0.63	0.63~0.7	>0.7
Eco-environment Subsystem A3	Eco-environmental water consumption rate(a14) (%)	Positive	<0.03	0.03~0.06	0.06~0.09	0.09~0.12	>0.12
	Wastewater treatment rate(a15) (%)	Positive	<94	94~96	96~97	97~99	>99
	Soil erosion rate (a16) (%)	Negative	>41	41~31	31~21	21~10	<10
	Forest coverage rate(a17) (%)	Positive	<17	17~30	30~42	42~54	>54
	COD emissions in wastewater (a18) (10,000 tons)	Negative	>511,500	511,500~388,200	388,200~264,900	264,900~141,500	<141,500

**Table 2:** The indicator system for sustainability assessment of water resources use.

Criterion Layer	Indicator Layer	Attribute	Weight
Water resource condition Subsystem A1 (0.465)	Annual precipitation (a1) (mm)	Positive	0.055
	Water resources per capita (a2) (m^3^)	Positive	0.306
	Water resources development and utilization rate (a3) (%)	Negative	0.011
	Water production modulus (a4) (10^4^ m^3^/10^6^ m^2^)	Positive	0.093
Socio-economic subsystem A2 (0.291)	Industrial water consumption rate (a5) (%)	Positive	0.053
	Agricultural water consumption rate (a6) (%)	Negative	0.046
	Household water consumption rate (a7) (%)	Positive	0.040
	Household wastewater emissions in urban area (a8) (10^4^m^3^)	Negative	0.010
	Water consumption per capita (a9) (m^3^)	Negative	0.009
	Water consumption per 10,000 yuan of GDP (a10) (m^3^/10,000 yuan)	Negative	0.010
	Water consumption per 10,000 yuan of industrial added value (a11) (m^3^)	Negative	0.017
	Cultivated land irrigation rate of (a12) (%)	Positive	0.066
	Effective utilization coefficient of cultivated land irrigation water (a13)	Positive	0.040
Eco-environment subsystem A3 (0.244)	Eco-environmental water consumption rate(a14) (%)	Positive	0.136
	Wastewater treatment rate(a15) (%)	Positive	0.021
	Soil erosion rate (a16) (%)	Negative	0.023
	Forest coverage rate(a17) (%)	Positive	0.049
	COD emissions in wastewater (a18) (10,000 tons)	Negative	0.015

**Table 3:** Cloud eigenvalues corresponding to five assessment grades of evaluation indices.

Indicator	Class I	Class II	Class III	Class IV	Class V
a1	(337.35, 138.13, 1)	(700, 169.85, 1)	(1075, 148.62, 1)	(1425, 148.62, 1)	(1796.8, 167.13, 1)
a2	(545.95, 419.58, 1)	(1535, 420.38, 1)	(2525, 420.38, 1)	(3515, 420.38, 1)	(66,708.6, 53,247.22, 1)
a3	(3.97, 1.34, 0.1)	(2.1, 0.25, 0.01)	(1.5, 0.25, 0.01)	(0.9, 0.25, 0.01)	(0.3, 0.25, 0.01)
a4	(14.95, 11.08, 1)	(41, 11.04, 1)	(67, 11.04, 1)	(93, 11.04, 1)	(119.08, 11.1, 1)
a5	(0.07, 0.05, 0.001)	(0.19, 0.05, 0.001)	(0.3, 0.04, 0.001)	(0.41, 0.05, 0.001)	(0.53, 0.05, 0.001)
a6	(0.8, 0.07, 0.001)	(0.65, 0.06, 0.001)	(0.49, 0.07, 0.001)	(0.33, 0.06, 0.001)	(0.17, 0.07, 0.001)
a7	(0.07, 0.04, 0.001)	(0.16, 0.04, 0.001)	(0.24, 0.03, 0.001)	(0.32, 0.03, 0.001)	(0.4, 0.04, 0.001)
a8	(585,217.5, 189,653.93, 1)	(317,850, 37,409.77, 1)	(229,800, 37,367.3, 1)	(141,750, 37,409.77, 1)	(53,658, 37,402.97, 1)
a9	(1593.05, 639.53, 1)	(770, 59.45, 1)	(600, 84.93, 1)	(425, 63.69, 1)	(265.95, 71.38, 1)
a10	(391.1, 34.9, 1)	(305, 38.22, 1)	(220, 33.97, 1)	(137.5, 36.09, 1)	(53.4, 35.33, 1)
a11	(101.95, 10.15, 1)	(80, 8.49, 0.1)	(60, 8.49, 0.1)	(40, 8.49, 0.1)	(18.9, 9.43, 0.1)
a12	(0.35, 0.08, 0.001)	(0.53, 0.08, 0.001)	(0.72, 0.08, 0.001)	(0.91, 0.08, 0.001)	(1.09, 0.08, 0.001)
a13	(0.47, 0.02, 0.001)	(0.54, 0.03, 0.001)	(0.6, 0.03, 0.001)	(0.67, 0.03, 0.001)	(0.72, 0.02, 0.001)
a14	(0.02, 0.01, 0.001)	(0.05, 0.01, 0.001)	(0.08, 0.01, 0.001)	(0.11, 0.01, 0.001)	(0.25, 0.11, 0.001)
a15	(93.4, 0.51, 0.01)	(95, 0.85, 0.01)	(96.5, 0.42, 0.01)	(98, 0.85, 0.01)	(99.65, 0.55, 0.01)
a16	(46.11, 4.34, 0.1)	(36, 4.25, 0.1)	(26, 4.25, 0.1)	(15.5, 4.67, 0.1)	(5.03, 4.23, 0.1)
a17	(10.94, 5.15, 0.1)	(23.5, 5.52, 0.1)	(36, 5.1, 0.1)	(48, 5.1, 0.1)	(60.4, 5.44, 0.1)
a18	(573,154.5, 52,360.51, 1)	(449,850, 52,356.69, 1)	(326,550, 52,356.69, 1)	(203,200, 52,399.15, 1)	(79,867, 52,342.25, 1)

**Table 4:** Results of sustainability assessment of water resource use in China.

Region	T-Comprehensive Assessment	C-Comprehensive Assessment	Assessment of Water Resource Condition Subsystem	Assessment of Socio-Economic SUBSYSTEM	Assessment of Eco-Environmental Subsystem
Beijing	Ⅳ	Ⅴ	Ⅰ	Ⅴ	Ⅴ
Tianjin	Ⅱ	Ⅴ	Ⅰ	Ⅳ	Ⅴ
Hebei	Ⅰ	Ⅰ	Ⅰ	Ⅱ	Ⅳ
Shanxi	Ⅰ	Ⅰ	Ⅰ	Ⅱ	Ⅱ
Inner Mongolia	Ⅰ	Ⅰ	Ⅱ	Ⅰ	Ⅴ
Liaoning	Ⅰ	Ⅰ	Ⅰ	Ⅱ	Ⅱ
Jilin	Ⅰ	Ⅱ	Ⅱ	Ⅰ	Ⅱ
Heilongjiang	Ⅰ	Ⅰ	Ⅴ	Ⅰ	Ⅰ
Shanghai	Ⅲ	Ⅴ	Ⅰ	Ⅴ	Ⅰ
Jiangsu	Ⅱ	Ⅰ	Ⅰ	Ⅲ	Ⅰ
Zhejiang	Ⅳ	Ⅴ	Ⅴ	Ⅲ	Ⅴ
Anhui	Ⅰ	Ⅰ	Ⅰ	Ⅲ	Ⅱ
Fujian	Ⅳ	Ⅴ	Ⅴ	Ⅲ	Ⅰ
Jiangxi	Ⅲ	Ⅴ	Ⅴ	Ⅱ	Ⅰ
Shandong	Ⅰ	Ⅰ	Ⅰ	Ⅱ	Ⅲ
Henan	Ⅰ	Ⅰ	Ⅰ	Ⅲ	Ⅳ
Hubei	Ⅰ	Ⅱ	Ⅱ	Ⅲ	Ⅰ
Hunan	Ⅲ	Ⅳ	Ⅳ	Ⅱ	Ⅰ
Guangdong	Ⅳ	Ⅴ	Ⅴ	Ⅲ	Ⅰ
Guangxi	Ⅲ	Ⅴ	Ⅴ	Ⅱ	Ⅰ
Hainan	Ⅱ	Ⅲ	Ⅲ	Ⅱ	Ⅰ
Chongqing	Ⅱ	Ⅱ	Ⅱ	Ⅳ	Ⅰ
Sichuan	Ⅰ	Ⅲ	Ⅳ	Ⅱ	Ⅰ
Guizhou	Ⅰ	Ⅲ	Ⅲ	Ⅰ	Ⅰ
Yunnan	Ⅰ	Ⅱ	Ⅲ	Ⅰ	Ⅰ
Tibet	Ⅴ	Ⅰ	Ⅴ	Ⅰ	Ⅰ
Shanxi	Ⅰ	Ⅱ	Ⅱ	Ⅱ	Ⅱ
Gansu	Ⅰ	Ⅰ	Ⅰ	Ⅰ	Ⅱ
Qinghai	Ⅰ	Ⅴ	Ⅴ	Ⅰ	Ⅱ
Ningxia	Ⅰ	Ⅰ	Ⅰ	Ⅰ	Ⅱ
Xinjiang	Ⅱ	Ⅰ	Ⅳ	Ⅰ	Ⅲ

The higher the grade, the darker the color, and the stronger the sustainability of water resources use in the region.

**Table 5:** Comprehensive evaluation indices and rankings of sustainability of water resources use and evaluation indices and rankings of subsystems by province and city in China.

Region	Comprehensive Level		Water Resource Condition Subsystem		Socio-Economic Subsystem		Eco-Environmental Subsystem	
	Index	Ranking	Index	Ranking	Index	Ranking	Index	Ranking
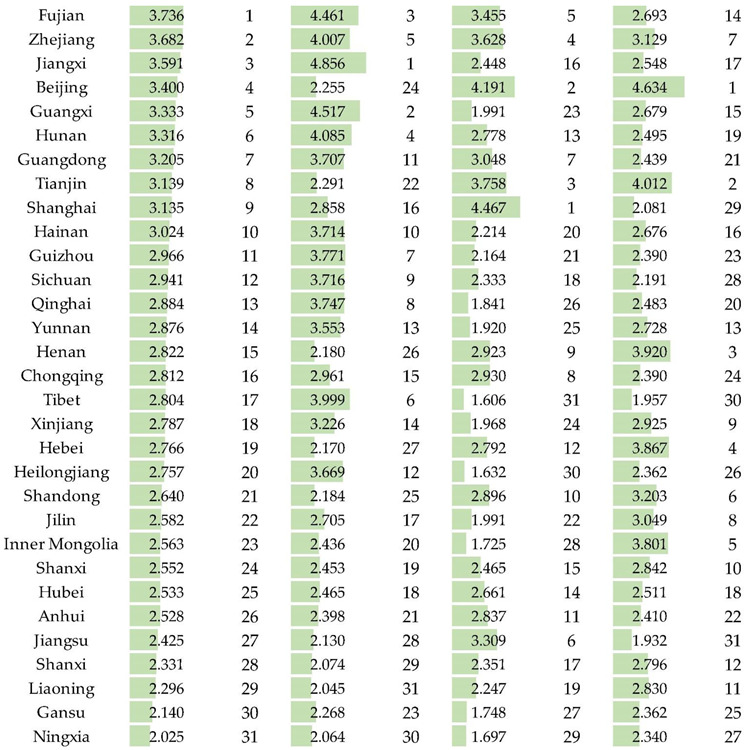								

The larger the evaluation index value, the longer the green column, and the stronger the sustainability of water resources use in the region.

## Data Availability

The relevant data can be found at the following websites: http://www.mca.gov.cn/ (accessed on 31 December 2019), http://www.mwr.gov.cn/sj/tjgb/zgstbcgb/ (accessed on 24 September 2020), http://www.mwr.gov.cn/sj/tjgb/szygb/ (accessed on 3 August 2020), http://www.stats.gov.cn/tjsj/ndsj/ (accessed on 1 September 2020 and 1 September 2021), http://www.shujuku.org/china-environment-statistical-yearbook.html (accessed on 1 December 2020).
